# Gender-Based Disparity in Academic Ranking and Research Productivity Among Canadian Anesthesiology Faculty

**DOI:** 10.7759/cureus.11443

**Published:** 2020-11-11

**Authors:** Eric N Esslinger, Michael Van der Westhuizen, Sabeena Jalal, Sarmad Masud, Faisal Khosa

**Affiliations:** 1 Anesthesiology, University of British Columbia, Vancouver, CAN; 2 Radiology, Vancouver General Hospital, Vancouver, CAN; 3 Anesthesiology, Shalamar Medical and Dental College and Hospital, Lahore, PAK

**Keywords:** academic promotion, h-index, academic anesthesiology, bibliometric analysis, gender disparity, gender equity, leadership, research productivity, academic rank

## Abstract

Purpose

Despite increasing numbers of women entering anesthesiology training, women remain underrepresented in senior academic positions and leadership roles. This study aims to determine the extent of gender disparity in Canadian departments of anesthesiology. In addition, we explore the correlation between publication productivity and academic rank in this cohort.

Methods

The Canadian Residency Matching Service (CaRMS) was queried to identify 17 training programs for anesthesiology. Department websites were searched to determine the names of faculty members, as well as gender, leadership roles, and academic ranks. The SCOPUS^©^ database was used to generate the number of publications, number of citations, publication range, and h-index of each faculty member.

Results

In our study cohort of 1404 academic anesthesiologists, 30.1% were women. Women held a minority of 130 leadership positions (27%, n = 35). With increasing academic rank female representation decreased (p = 0.009), such that 21% of full professors were women. Overall, male anesthesiologists had a higher h-index, number of publications, and number of citations (p = 0.001, p = 0.001, and p = <0.001, respectively) than women.

Conclusion

Despite growing numbers of women entering the academic workforce, women are underrepresented in senior academic ranks and leadership positions. In addition, men and women have significant differences in measures of publication productivity. This study underscores the importance of directed efforts to promote equity in career outcomes.

## Introduction

It is now two decades since medical school enrollment reached equal numbers of men and women [1]. Following the rise of female medical students, there has been a concomitant increase in the number of women entering the field of anesthesiology. Women now represent 33% of Canadian anesthesiologists, which is a dramatic improvement from 25% in 2005, and 22% in 1998 [2,3]. The latest data show that 42% of anesthesiologists aged less than 35 are women [3]. Canadian Residency Matching Service (CaRMS) data show that women are as likely as men to successfully obtain their top choice residency program, suggesting that equity has been achieved at the level of entering the specialty [4]. Improved gender diversity has assuredly impacted anesthesiology in a positive manner, as women physicians are known to spend more time counselling patients, better follow guidelines, and have lower mortality and readmission rates [5-7].

Despite growing numbers of women entering the specialty, gender disparity is still pervasive. Within the academic realm, women secure fewer first authorships, receive less funding, and have lower editorial board membership [8-10]. In 2018, the Canadian Anesthesiologist's Society (CAS) Honorary Award winners were universally men [11]. In addition, anesthesiology leadership remains predominately male. In the 77-year history of the CAS, only three out of 67 presidents were women [12]. Of 17 Canadian departments of anesthesiology, three are currently chaired by women.

Factors that undermine female advancement in academic anesthesiology are complex and incompletely understood. Previous research has highlighted several common factors: a lack of female mentors, disproportionate family responsibilities, and gender biases (which affect hiring, promotion, and career trajectory) [13]. Research productivity correlates strongly with academic promotion; however, it is not known how research output relates to gender for Canadian anesthesiologists [14].

The objective of this study is to examine the gender balance of Canadian anesthesiologists in academic settings and the associations with academic rank, leadership roles, and research productivity. By evaluating these factors, this study aims to better understand the extent of, and influences for, gender disparity among academic anesthesiologists.

## Materials and methods

This retrospective, cross-sectional study examined the gender distribution of academic anesthesiologists in Canadian university departments and analyzed the associations of gender, academic rank, leadership roles and research productivity. Our methodology has been validated in recent publications [15-17]. This study did not require institutional ethics board approval as all data were available from public websites.

Data collection

The CaRMS website was consulted to generate a list of 17 university affiliated programs that offer anesthesiology residency training in Canada. Of these programs, 15 provided listings of faculty members on department websites and were included in the study. Our inclusion criteria were faculty members who were physicians (MD or MD/PhD) and received residency training from an accredited Canadian program, and for whom academic rank and leadership information was available. 1404 individuals met criteria for inclusion. Data extracted from department websites included name, academic rank (if any), and leadership position (if any). Gender was obtained from provincial college registration, or as agreed upon by all authors from name and faculty photo. Academic ranks were either Assistant Professor, Associate Professor, or Clinical (Full) Professor. Leadership roles were defined as any Director, Dean, Department Head, Chief, or any Committee Chair, Vice-Chair, or Co-Chair. Northern Ontario School of Medicine did not list anesthesiology faculty and University of Saskatchewan did not list academic ranks and therefore these two schools were excluded from analysis. Throughout data collection, errors of duplications and omissions due to name changes were mitigated by searching multiple sources for information, including Doximity and LinkedIn where information was incomplete. Data was collected in 2017.

For each faculty member, SCOPUS^©^ was queried to gather data regarding research productivity. Parameters recorded were number of documents published, total number of citations, year of first publication, year of most recent publication, and Hirsch (h)-index. H-index is a bibliometric that quantifies research productivity. Its calculation incorporates the number of publications and number of citations to provide a quantitative and qualitative measure of an author’s research performance. An inherent limitation of this index is that it overestimates performance of senior researchers as they have a longer duration to accrue publications and citations [18]. The SCOPUS^©^ database was chosen for bibliometric analysis as it has been found to have a greater scope of coverage and more reliable h-index calculation than other medical literature databases [19].

Analysis

All analyses were performed using SPSS software (IBM Corp., Armonk, NY). Continuous variables (h-index, citations, publications, years of research) were tested for normality and log transformation was done. All continuous variables were skewed in distribution. Therefore, non-parametric analysis (Mann-Whitney U test) was applied to continuous variables to assess male versus female differences. Statistical significance was defined as p < 0.05 in all analyses. A multigression analysis was performed to build a final model to predict h-index. At the univariate level simple linear regression was applied. Each variable was regressed independently with h-index, their assumptions were checked, and their significance was reported. Independent variables were checked for multi-collinearity and with presence defined as a correlation coefficient of 0.8. There was no multi-collinearity seen. Cramer’s V test was used for one nominal and one ordinal variable, and Spearman test was used for one continuous variable and one ordinal variable.

Main effects were identified using stepwise selection strategy and based on the p value, from which we decided to include a variable in the model or drop it. The final step was to check for interaction. Interaction terms were created between each of the main effects in the model and there was no significant interaction. ‘Academic rank’, ‘Publications’, and ‘Citations’ were not confounders for h-index.

## Results

Our methods identified 1404 anesthesiologists, of whom 982 (69.9%) were males and 422 (30.1%) were females. A minority of faculty positions were held by women in all provinces with a range from 22.9% (British Columbia) to 36.5% (Quebec) (Table 1). No significant association was observed when comparing faculty membership and gender (χ^2^ = 0.66; p = 0.416).

**Table 1:** Distribution of men and women anesthesiology faculty in academic institutions across Canada

	Men		Women	
Province	Frequency	Percent	Frequency	Percent
Alberta	200	72.46	76	27.54
British Columbia	54	77.14	16	22.86
Manitoba	83	73.45	30	26.55
Newfoundland	41	74.55	14	25.45
Manitoba	103	67.76	49	32.24
Ontario	353	69.9	152	30.1
Quebec	148	63.52	85	36.48
Total	982	69.94	422	30.06

**Figure 1:**
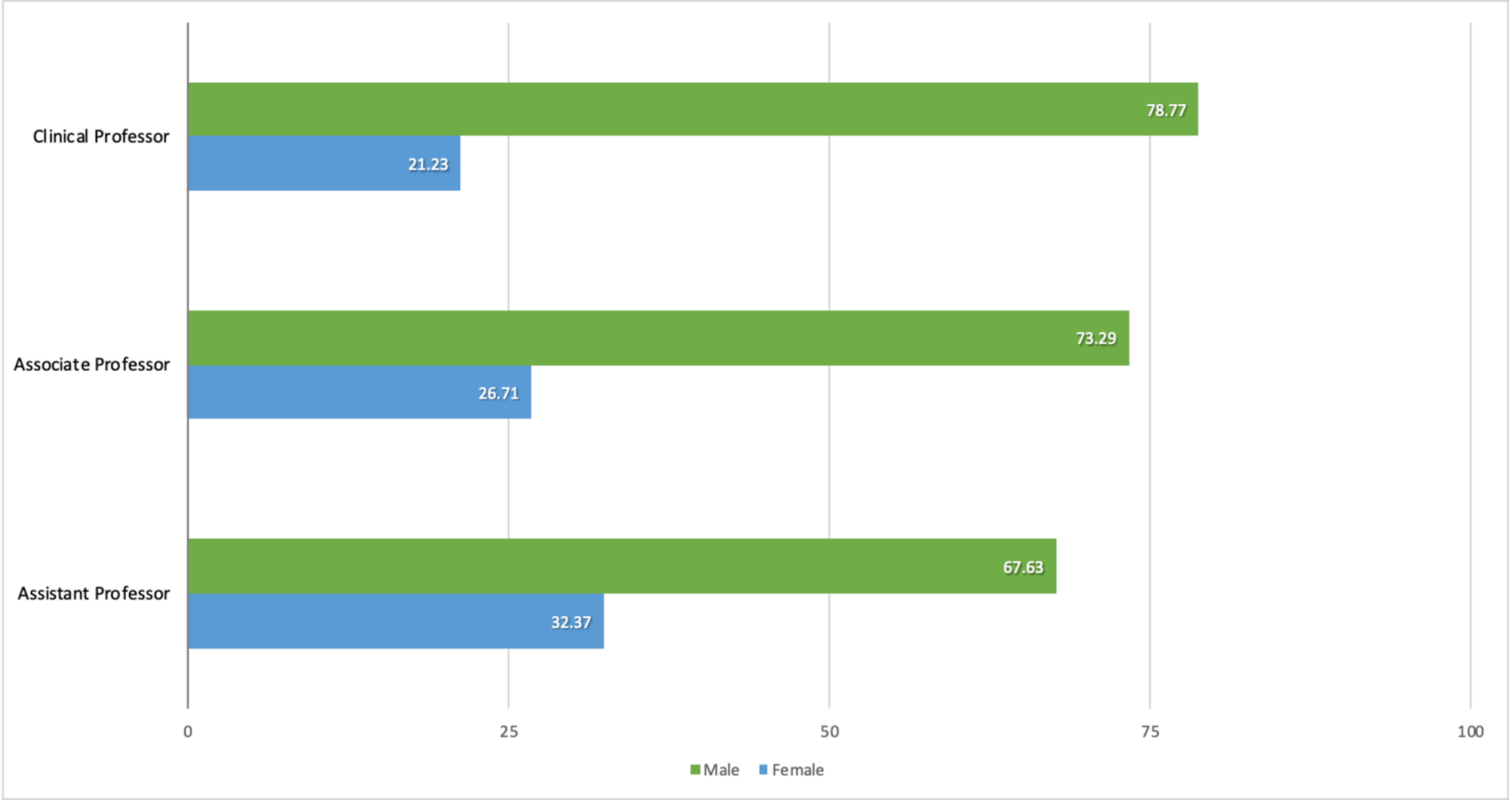
Distribution of men and women anesthesiology faculty members by academic rank. The data show a linear trend with an increasing percentage of men at each successive rank. Men and women differ significantly in their distribution (p = 0.009). Numbers represent percentages of men or women at an academic rank.

Analysis of academic ranks revealed a trend for decreasing female representation with increasing academic rank. Women accounted for 32% of assistant professors, 27% of associate professors, and 21% of clinical professors (Figure 1). Only 7.3% of female faculty held the top professorship level, which was a lower proportion than that of men (11.7%). We found a significant difference in the distribution of men and women across academic ranks (χ^2^ = 9.42; p = 0.009).

Leadership positions were also disproportionately held by men. Overall, 9.2% (n = 130) of faculty members held leadership positions (Table 2). Of these, men held 73% (n = 95) whereas women held 27% (n = 35).

 

**Table 2:** Distribution of men and women anesthesiology faculty members according to academic rank and leadership position

Rank	Men	Women	Total
All academic faculty members	983 (69.9%)	422 (30.1%)	1404 (100%)
Academic rank			
Assistant Professor	654 (67.6%)	313 (32.4%)	967 (100%)
Associate Professor	214 (73.3%)	78 (26.7%)	292 (100%)
Clinical Professor	115 (78.8%)	31 (21.2%)	146 (100%)
Leadership position			
No position	888 (69.7%)	387 (30.4%)	1275 (90.8%)
Leadership position	95 (73.1%)	35 (26.9%)	130 (9.2%)

The median number of publications, citations, and years of research were tabulated, and significant gender differences were determined by Mann-Whitney U-test (Tables 3, 4, 5). Overall, men authored significantly more publications than women (median 17 vs 10, p = 0.001). In addition, men had a significantly higher number of citations (median 428 vs 272, p = 0.01) and years of research (median 10 vs 7, p <0.001). The values for each variable rose with increasing academic rank in both genders. Of note, women in leadership positions had more publications (10 vs 7), citations (161 vs 122) and more than twice as many years of research (22 vs 10) in comparison to their male counterparts. Women in leadership roles had equivalent h-index to men.

**Table 3:** Median number of publications of men and women faculty members stratified by academic rank and leadership position. Range shown in brackets.

Rank	Number of publications	
	Men	Women
Academic rank		
Assistant Professor	4 (1-91)	2 (1-79)
Associate Professor	9 (1-237)	10 (1-122)
Clinical Professor	67 (1-261)	29.5 (1-387)
Leadership position		
Holds leadership position	7 (1-233)	10 (1-81)
Total	17 (0-261)	10 (0-387)

**Table 4:** Median number of citations of men and women faculty members stratified by academic rank and leadership position. Range shown in brackets.

Rank	Number of citations	
	Men	Women
Academic rank		
Assistant Professor	79 (0-3698)	52 (0-3113)
Associate Professor	258 (0-8342)	202.5 (4-3408)
Clinical Professor	1473 (0-24614)	792 (24-13594)
Leadership rank		
Holds leadership position	122 (0-8342)	161 (2-3113)
Total	428 (0-24614)	272 (0-13594)

**Table 5:** Median years of research for men and women faculty members measured by publication range. Range shown in brackets.

Rank	Years of research	
	Male	Female
Academic rank		
Assistant Professor	6 (1-58)	2 (1-33)
Associate Professor	13 (1-49)	15 (1-40)
Clinical Professor	27 (1-42)	29 (1-45)
Leadership rank		
Holds leadership position	10 (1-34)	22 (1-45)
Total	10 (0-58)	7 (0-45)

The h-index, a metric incorporating citation and publication counts to assess publication productivity, was assessed for the cohort of 1404 anesthesiologists. The lowest indices were seen in assistant professors, with men and women both having a median of 3 (Table 6). The highest indices were seen in full professors, with men having a median of 20 (range 0-56), and women 11.5 (2-63). Similar to citation and publication count a positive relationship between h-index and rank was seen in both genders. H-index values were comparable at the Assistant and Associate Professor level, however male Clinical Professors had a significantly higher h-index (p = 0.002).

**Table 6:** Median h-index of men and women anesthesiology faculty members stratified by academic rank and leadership position. Range shown in brackets.

Rank	H-index	
	Men	Women
Academic rank		
Assistant Professor	3 (0-25)	3 (0-26)
Associate Professor	6.5 (0-45)	7 (1-28)
Clinical Professor	20 (0-56)	11.5 (2-63)
Leadership position		
Holds leadership position	5 (0-50)	5 (1-24)
Total	5 (0-56)	3 (0-63)

A multivariate analysis was conducted to build a model to predict h-index. Univariate regression identified variables that were significant with h-index, which were ‘Gender’, ‘Publications’, ‘Citations’, ‘Years of active research’ and ‘Academic rank’. Variables ‘Leadership rank’ and ‘Province’ were dropped from the model as they were insignificant.

The final model was: y(x) = β0 + β1 (Female) + β2 (Academic ranks) + β3 (Years of Active Research) + β4 (Publications) + β5 (Citations)

This prediction equation accounted for major variability in the model as adjusted R square = 0.8992, F test was 1218.70, and p-value was ≤ 0.001. Our model found that female faculty have 0.78 times the odds of having a higher h-index than male faculty, keeping all other variables constant.

## Discussion

In this study, we report on the status of men and women in Canadian departments of anesthesiology. While we found the proportion of women in academic departments approaches that of the medical workforce (30.1% vs. 33%, respectively), women are significantly underrepresented in top academic ranks [3]. With increasing rank, female representation declined, such that women account for 32% of assistant professors, 27% of associate professors, and only 21% of clinical professors (Figure 1). The academic rank difference between male and female anesthesiologists was statistically significant (p=0.009). Only 7% of women in this study attained full professorship, compared to 12% of men. These results are similar to those found in the United States, where 7.4% of women faculty members were full professors, versus 17.3% of men [20]. Historical US datasets show that the proportion of women reaching full professorship rose only 0.9% in a decade, suggesting stagnation [21]. As this cross-sectional study represents a snapshot in time, future studies are needed to determine trends in Canada.

Research output is a key factor for promotion in academic institutions [14]. To quantify the academic productivity of an individual researcher, h-index is widely used [18]. Our multivariate analysis of h-index found that women have 0.78 times the odds of having a higher h-index than men, holding all other variables constant. Furthermore, women had significantly lower publication counts (10 vs. 17, p=0.001), citation counts (272 vs. 428, p = 0.01), and fewer years of research (7 vs. 10, p<0.001) than men. In both genders, higher values of these metrics correlated with increased academic rank (Tables 3, 4, 5). 

Positions of leadership, such as department chair or dean, were analyzed in our cohort of 1404 academic anesthesiologists. While one in 10 faculty members held a formal leadership position, disproportionately fewer (27%) were held by women. This pervasive gender imbalance is cause for concern. Because chairs, department heads, and other leadership roles carry influence in allocating funds, policymaking, and advocacy, women may have a lesser role in directing healthcare. Equity, diversity, and inclusion (EDI) within anesthesiology organizations is critical not only for proportionate representation in leaders, but because high-quality healthcare is achieved by leaders with diverse backgrounds, skillsets, and ideas. Recruitment appears to be a barrier with few women entering applicant pools. In a reflection of 25 years of hiring practices in academic medicine, Rochon suggests that women less frequently state interest in academic positions, are reticent from lower perceived self-efficacy, and prioritize other commitments, such as childcare [22]. Systemic barriers, including deeply-engrained unconscious gender bias and a lack of mentors likely contribute as well [23]. We find that women leaders are successful scholars with more publications, citations, and a longer research career than their male counterparts (Tables 3, 4, 5). Initiatives aimed at engaging women in academics represent a potential solution for the gender gap identified.

There are a number of reasons why fewer women are found in top academic ranks. Our findings suggest that lower research productivity among women is a potential factor. Prior work has shown that women may have lower research output because they are concentrated in the youngest age groups [3]. Indeed, a US study found that female anesthesiologists have lower early-career research productivity than men [14]. Canadian data shows that female anesthesiologists of child-rearing age have lower workloads than men and may get fewer research and leadership experiences during early career development [24]. In addition, women returning from maternity leave may not take on academic duties [25].

Several barriers to female advancement in academic medicine have been proposed. Women take on a disproportionate amount of family responsibilities, and these commitments take away time needed for research projects [24]. Women are hindered by unconscious gender bias during selection and promotion for academic positions [26]. For example, male names are ascribed greater competence on applications [27]. Mentorship is important for fostering research skills and interest in trainees; however, female role models and mentors are scarce in anesthesiology [28]. Finally, women with family commitments are disadvantaged by organizational practices, such as inflexible schedules and meeting times [23].

The gender gap in academic anesthesiology has not been corrected by growing numbers of female trainees. Through this study, we hope to take an important step toward change by recognizing that women are critically underrepresented in academia and leadership. Solutions should take place at multiple levels, such as fostering career interest in medical students, providing mentors for trainees, and creating leadership opportunities for junior faculty [24,29]. Institutions and organizations need to consider the contributions of women when selecting board members, journal editors, award recipients, and professors [30]. In light of our findings that women have lower measures of research metrics, we suggest that all female anesthesiologists gain research experience early in their careers. Early academic undertakings can extend into future research opportunities as well as a growing body of publications and citations for academic promotion.

There are several limitations to this research. There are potential inaccuracies in faculty listings from institutional websites as they may have been outdated when accessed. Despite efforts made to cross-reference names, errors of duplication or omission may have occurred with name changes. Furthermore, name changes from marriage or divorce disproportionately affect women. In addition, data from the University of Saskatchewan and Northern Ontario School of Medicine were not available and therefore our results are not representative of all anesthesiology faculty in Canada. Lastly, there are inherent limitations with h-index, such as a bias toward researchers with longer careers, and the inability to distinguish author order or self-citation [18].

## Conclusions

This study found significant gender disparity in academic anesthesiology in Canada. Despite a growing presence of women in anesthesiology, men are overrepresented in top academic and leadership positions. The data demonstrated an association between research metrics and academic rank, and women were found to score lower in these metrics than men. Targeted initiatives and policies, such as early-career research opportunities for women, should be initiated to promote equity, diversity, and inclusion within the anesthesiology workforce.
